# Hypoxia‐induced activin A diminishes endothelial cell vasculogenic activity

**DOI:** 10.1111/jcmm.13306

**Published:** 2017-08-18

**Authors:** Stephanie Merfeld‐Clauss, Hongyan Lu, Xue Wu, Keith L. March, Dmitry O. Traktuev

**Affiliations:** ^1^ Department of Medicine Division of Cardiology Indiana Center for Vascular Biology and Medicine Krannert Institute of Cardiology Indianapolis IN USA; ^2^ VA Center for Regenerative Medicine R.L. Roudebush VA Medical Center Indianapolis IN USA; ^3^ Department of Medicine Division of Hematology/Oncology Indiana University School of Medicine Indianapolis IN USA; ^4^ Department of Cellular and Integrative Physiology Indiana University School of Medicine Indianapolis IN USA

**Keywords:** endothelial cells, activin A, coculture, vasculogenesis, hypoxia inducible factor, adipose stem/stromal cells

## Abstract

Acute ischaemia causes a significant loss of blood vessels leading to deterioration of organ function. Multiple ischaemic conditions are associated with up‐regulation of activin A, but its effect on endothelial cells (EC) in the context of hypoxia is understudied. This study evaluated the role of activin A in vasculogenesis in hypoxia. An *in vitro* vasculogenesis model, in which EC were cocultured with adipose stromal cells (ASC), was used. Incubation of cocultures at 0.5% oxygen led to decrease in EC survival and vessel density. Hypoxia up‐regulated inhibin B_A_ (monomer of activin A) mRNA by 4.5‐fold and activin A accumulation in EC‐conditioned media by 10‐fold, but down‐regulated activin A inhibitor follistatin by twofold. Inhibin B_A_ expression was also increased in human EC injected into ischaemic mouse muscles. Activin A secretion was positively modulated by hypoxia mimetics dimethyloxalylglycine and desferrioxamine. Silencing HIF1α or HIF2α expression decreased activin A secretion in EC exposed to hypoxia. Introduction of activin A to cocultures decreased EC number and vascular density by 40%; conversely, blockade of activin A expression in EC or its activity improved vasculogenesis in hypoxia. Activin A affected EC survival directly and by modulating ASC paracrine activity leading to diminished ability of the ASC secretome to support EC survival and vasculogenesis. In conclusion, hypoxia up‐regulates EC secretion of activin A, which, by affecting both EC and adjacent mesenchymal cells, creates a micro‐environment unfavourable for vasculogenesis. This finding suggests that blockade of activin A signalling in ischaemic tissue may improve preservation of the affected tissue.

## Introduction

Efficient revascularization of ischaemic tissues is essential for sufficient functional recovery of tissues after acute injuries, including myocardial infarction, brain and kidney insults and burns. Inadequate tissue vascularization leads to loss of function, fibrosis and development of non‐healing wounds seen after radiation therapy and in patients with diabetes or peripheral artery diseases. Defining the responses of the vasculature to severe hypoxia is important to understand the natural recovery of affected tissues after blood flow has been restored. It is also important for understanding the survival of tissue‐engineered grafts following implantation, and following implantation of vasculogenic cell mixtures, composed of both endothelial and mesenchymal stromal cells, directly into ischaemic tissues, immediately after their exposure to hypoxia. Low oxygen tension may have profound effects on the ability of endogenous as well as implanted cells to reorganize into functional vasculature.

Up‐regulation of activin A expression and secretion has been previously observed in infarcted myocardium 1, 2, 3, 4, 5, ischaemic brain 6, 7 and kidney 8, burns 9, and following mechanically damaged to the cornea 10. Most of these disorders are associated with local hypoxia, accumulation of reactive species and inflammation. In addition, an increase in serum activin A concentration has been reported in patients with heart failure 4, where its level correlated with the stage of disease, as well as in patients with other pathological conditions, including hyperthyroidism, liver cirrhosis and renal failure 11, and pre‐eclampsia, which is associated with an increase in activin A level in maternal blood 12. Furthermore, a correlation has been described between the degree of foetal hypoxia and activin A level in cord blood 13.

Currently, most of the data relevant to the effects of activin A on EC biology have been obtained from *in vitro* studies, and in many cases have been conflicting. Several studies have shown that activin A has anti‐angiogenic activity, including suppression of EC growth 14, 15 and attenuation of vasculogenesis in Matrigel assays 14. Other studies have shown that activin A augments the effect of vascular endothelial growth factor (VEGF) on EC proliferation and tubulogenesis 16, 17. In parallel, follistatin, natural inhibitor of activin A, is able to induce angiogenesis 18. Our recent studies suggest that activin A plays an important role in communication of two complementary cell types, endothelial and ASC, engaged in the process of vasculogenesis. We have shown that activin A is induced in ASC in response to direct contact with EC and stimulates differentiation of progenitor mesenchymal cells towards mural cell phenotype 19, thus promoting formation of multilayered vessels. In parallel, it transforms the ASC secretome from pro‐angiogenic towards angiostatic net bioactivity 19, thus shifting the vasculogenic process from the initiation to a resolution phase and orchestrating the formation of mature stable vessels. In these studies, the primary source of activin A was identified to be the ASC, whereas its expression in EC did not change during the vasculogenic process.

Interestingly, in studies to date, the induction of activin A has been reported specifically only in parenchymal cells, such as renal tubular cells 8, cardiomyocytes 4 and neurons 7, 20, whereas no data are available with respect to its expression in the vasculature of the affected tissues. It is not clear if this is due to the relative difficulty of detecting activin A in EC or indeed absence of its expression in EC in the ischaemic conditions analysed.

This study was designed to evaluate the effect of activin A on hypoxia‐exposed vasculature and includes testing EC ability to undergo vasculogenesis in hypoxic environments, assessing the effects of hypoxia on expression of activin A by EC and ASC and defining the linkage between these two processes using the *in vitro* model of EC cocultivation with ASC which we have previously described 21.

## Materials and methods

### Isolation and culture of cells

All procedures for collecting umbilical cord and adipose tissue were approved by the Indiana University School of Medicine Institutional Review Board.

#### Human ASC

Human ASC were isolated from human subcutaneous adipose tissue samples obtained from liposuction procedures as previously described 19. Samples were digested in 1 mg/ml of collagenase type I solution (Worthington Biochemical, Lakewood, NJ, USA) for one hour at 37°C and centrifuged at 300 *g* for 8 min. to separate the stromal cell fraction (pellet) from adipocytes. The pellets were filtered through 250 μm Nitex filters (Sefar America Inc., Kansas City, MO, USA) and treated with red cell lysis buffer (eBiosciences, San Diego, CA, USA). The final pellet was resuspended and cultured in EGM‐2MV (Lonza, Walkersville, MD, USA). Media were changed after 24 hrs and then every 2–3 days. ASC were passaged when 60–80% confluent and used at passages 3–5.

#### Cord blood derived endothelial cells (CBD‐EC)

Cord blood derived endothelial cells were isolated from the umbilical cord vein blood of healthy newborns (38–40 weeks gestational age) as previously described 22. Mononuclear cells were isolated from blood by gradient centrifugation through Histopaque 1077 (ICN, Costa Mesa, CA, USA) and cultured in EGM‐2/10%FBS (Lonza) in tissue culture plates pre‐coated with 50 μg/ml of rat tail collagen type I (BD Biosciences, San Diego, CA, USA). Culture medium was changed daily for 7 days and then every other day until first passage. Cells were passaged when 90% confluent and used at passages 4–6.

Human umbilical vein EC (HUVEC), human cardiac microvascular EC (HMVEC), human retinal endothelial cells (HREC) were purchased from Lonza, expanded in EGM‐2MV media and used at passages 5–7.

### *In vitro* models of vasculogenesis

#### Two‐dimensional model

6 × 10^4^ ASC/cm^2^ and 5 × 10^3^ EC/cm^2^ were mixed in EBM‐2/5%FBS and plated on cell culture plastic. To evaluate the effect of hypoxia on vascular network formation, cocultures were incubated at 21% O_2_ (standard conditions) or at 0.5–5% O_2_ for up to 9 days with media exchange every third day. To challenge cells with low oxygen tension, plates were placed into Pro‐Ox C21/C‐Chamber (BioSpherix, Lacona, NY, USA). To evaluate the role of activin A in EC vasculogenesis, cocultures were incubated for the first 3 days of experiments in: (*i*) control media alone or supplemented with either 25 ng/ml of activin A or 10 μg/ml activin A neutralizing IgG, or isotype control IgG (R&D Systems, Minneapolis, MN, USA); (*ii*) 3.5‐fold concentrated media conditioned by EC for 72‐hr in normoxia or hypoxia, either unmodified or pre‐treated with 10 μg/ml activin A neutralizing IgG or isotype control IgG.

#### Three‐dimensional model

6 × 10^5^ ASC and 6 × 10^5^ DsRed‐expressing EC were mixed and resuspended in fibrinogen at final concentration 2.5 mg/ml, and 60 μl of cell solution was added to the wells of 96‐well plates containing 3 μl of 10 U/ml thrombin. Polymerized gels were overlaid with EBM‐2/5%FBS and incubated in standard conditions for 48 hrs, followed by dividing plates between incubators with 5% and 21% O_2_ for additional 72‐hr incubation. At day 5, vascular networks were imaged by fluorescent microscopy. Subsequently, plates, incubated in standard conditions during all prior steps, were exposed to 5% O_2_ for 72 hrs, followed by image acquisition.

### Immunohistochemical culture evaluation

To evaluate vascular network formation, cocultures were fixed in methanol (−20°C for 5 min.) and probed with biotinylated Ulex Europaeus Agglutinin I (Vector labs) for 1 hr, followed by incubation with Streptavidin Alexa 488 (Invitrogen) USA for 30 min. The nuclei were counterstained with DAPI. Fluorescently stained cultures were imaged using a Nikon Eclipse Ti microscope. Digital images were acquired using a 4x objective (9 pictures/cm^2^ = 30% of the well surface). Images of vascular networks were processed by the MetaMorph software ‘Angiogenesis Tube Assay’ algorithm (Molecular Devices, Downingtown, PA, USA).

### Cell suspension analysis by flow cytometry

EC‐ASC cocultures were harvested, cell numbers were determined using hemocytometer and cell suspensions were incubated with CD140b‐PE (ASC marker) and CD31‐APC (EC markers) IgG (BD, San Diego, CA, USA) on ice for 20 min. ASC:EC ratios were determined using Calibur flow cytometer and Cell QuestPro software (BD).

### Total RNA and conditioned media (CM) generation

6 × 10^4^ ASC or EC per cm^2^, or 6 × 10^4^ ASC admixed with 3 × 10^4^ EC per cm^2^, were incubated in EBM‐2/5%FBS in standard conditions or at 0.5% O_2_ for 24–72 hrs. Cells were lysed at selected time‐points, and total RNA was isolated using NucleoSpin RNA II kit (Clontech, Mountain View, CA, USA). In parallel, media conditioned by the cells were collected after 48‐ and 72‐hr incubation. Cells were counted using a hemocytometer at each media collection time. For subsets of experiments, EC‐CM were 10‐fold concentrated using Amicon Ultra‐15 Ultracell 3K columns (MilliporeTemecula, CA, USA). Another set of CM was generated by incubating EC in standard conditions in the presence of 1 mM dimethyloxalylglycine (DMOG), a cell permeable prolyl‐4‐hydroxylase inhibitor, or 500 μM desferrioxamine (DFO), an iron chelator, or DMSO, a solvent control, for 24 hrs (all from Sigma‐Aldrich St. Louis, MO, USA).

To evaluate role of hypoxia inducible factors (HIFs) in modulation of activin A secretion, EC were transduced with lentiviral constructs encoding shRNA for HIF1α or HIF2α (gift from Mircea Ivan) and also expressing puromycin resistance gene. Transduced EC, after antibiotic selection, were expanded and plated for media conditioning in normoxic or hypoxic conditions.

To assess the role of activin A in ASC bioactivity, ASC were incubated in EBM‐2/5%FBS alone or supplemented with 25 ng/ml of activin A for 48‐hrs followed by CM collection and cell lysis for RNA isolation.

CM were evaluated for presence of activin A by ELISA (R&D Systems).

### Analysis of human Inhibin B_A_ expression by EC in mouse ischaemic muscle

Animals were cared for in accordance with guidelines published by NIH, and all study procedures were approved by the Indiana University IACUC. Unilateral hindlimb ischaemia was created in male NOD/SCID/IL2Rg null mice (Indiana University animal core, 8 months old) as described previously 23. The animals were anaesthetized with 2.5% isoflurane after which an incision was made at the midline of the left hindlimb. The femoral artery, vein and their branches were ligated, beginning at the inguinal ligament to the bifurcation of saphenous and popliteal arteries, followed by excision of the region between the ligatures. Three hours after surgery, 2.5 × 10^5^ EC in 10 μl of media were injected into *m. tibialis anterior* of both left (ischaemic) and right (intact) limbs. Twenty‐four hours post‐cell injection, muscles were harvested, total RNA was isolated and the level of inhibin B_A_ mRNA expression was evaluated by quantitative PCR using human‐specific primers.

### Evaluation of EC and ASC antigen expression by qRT‐PCR

cDNA was generated from total RNA using iScript RT kit, and polymerase chain reactions (PCR) were performed with iTaq SYBR Green PCR Supermix (Bio‐Rad, Hercules, CA, USA). PCR were performed in 25 μl reaction mix containing 5 ng cDNA, 400 nM of each primer. Thermal cycling parameters were as follows: 3 min. at 95°C for polymerase activation followed by 40 cycles of 10 sec. at 95°C for denaturation and 30 sec. for anneal/extend. Oligonucleotides used as primers are presented in Table 1. TaqMan reactions with primers/probe specific to human β‐actin (Invitrogen) were performed for normalization of total human EC presence in the mouse muscle tissues.

**Table 1:** Sequence of primers used for PCR reaction

Antigen	Sense strand	Antisense strand	T_A_
Inhibin B_A_	GGAGAACGGGTATGTGGAGA	AATCTCGAAGTGCAGCGTCT	62°C
Follistatin	CTTTGCCTCCTGCTGCTG	ACTCCTCCTTGCTCAGTTCG	62°C
ALK4	TCAGAAGCTGCGTCCCAACATC	CGTTGGCATACCAACACTCTCG	62°C
β‐Actin	CACCATTGGCAATGAGCGGTTC	AGGTCTTTGCGGATGTCCACGT	62°C

### EC transfection with inhibin B_A_ siRNA constructs

EC were transfected with a mixture of the two most potent inhibin B_A_ siRNA constructs or scrambled siRNA (scRNA) (Origene, Rockville, MD, USA) using Lipofectamine RNAiMAX reagent (Invitrogen). On the following day, cells were harvested and used for coculture experiments. Preliminary tests confirmed 80–95% decrease in expression of activin A post‐transfection.

### EC proliferation assay

EC were plated at 5 × 10^3^ cells/cm^2^ in EBM‐2/5%FBS. On the following day, media were exchanged to: (*i*) EBM‐2/5%FBS alone or with 25 ng/ml activin A; (*ii*) 50% CM (48‐hr) collected from control ASC alone or with 25 ng/ml activin A; and (*iii*) 50% CM (48‐hr) collected from ASC incubated in the presence of 25 ng/ml activin A. Three days later, cells were fixed, stained with DAPI and quantitatively evaluated.

### Western blotting

EC, plated at 5 × 10^4^ cells/cm^2^, were incubated in EBM‐2/5%FBS media alone or supplemented with either 500 μM DFO or 1 mM DMOG for 24 hrs Also, EC expressing shRNA either for HIF1α or for HIF2α were incubated in control media either in normoxia or hypoxia for 40 hrs. After incubation, cells were lysed with 1%SDS/PBS lysis buffer containing cocktail of protease inhibitors. Proteins were fractionated on 4–20% sodium dodecyl sulphate–polyacrylamide gel by electrophoresis and transferred onto 0.45 μm nitrocellulose membranes. Membranes were incubation with HIF‐1α, HIF‐2α (RnD Systems) or β‐actin (Sigma‐Aldrich) IgG, followed by incubation with peroxidase‐conjugated goat antimouse IgG (Cell Signaling Danvers, MA, USA). The bands were visualized using Western Lightning Chemiluminescence Reagent Plus (Perkin Elmer American Fork, Utah, USA ).

### Computational and statistical analysis

Fluorescently stained cultures were analysed using a Nikon TE2000 microscope. Digital images were acquired using a 4x objective. Quantitative data are expressed as mean ± S.E.M. Statistical analysis of data including only two experimental groups was performed with an unpaired t‐test. Analysis of the data with at least three groups was performed with one‐way ANOVA with Tukey's multiple comparisons test. Statistical analysis was performed with Prism 4 (Graphpad, San Diego, CA, USA).

## Results

### Effect of hypoxia on vascular network formation (VNF)

**Figure 1:**
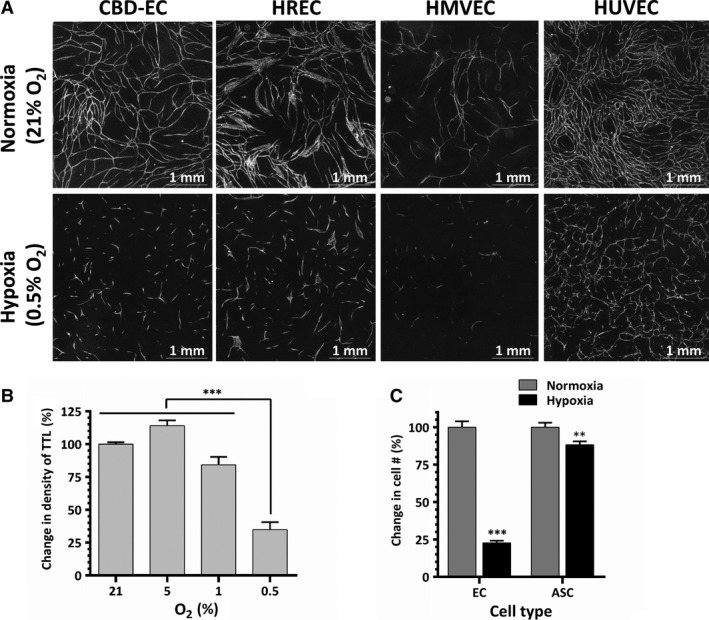
(**A**) Representative images of vascular networks formed by EC from cord blood (CBD‐EC), retinal (HREC) and cardiac microvasculature (HMVEC), and umbilical vein (HUVEC) on ASC monolayer. Cocultures were incubated in EBM‐2/5%FBS in normoxia (21%O_2_) or hypoxia (0.5%O_2_). Images were taken at day 6 post‐plating. (**B**) Comparative quantitative analysis of VNF (density of total tube length, TTL) by CBD‐EC on ASC when cultured in EBM‐2/5%FBS and exposed to normoxia (21%O_2_) or hypoxia (0.5–5%O_2_) for 6 days (*n* = 4–30). (**C**) Comparative analysis of CBD‐EC and ASC survival in EC‐ASC cocultures after incubation in normoxia or hypoxia. At day 6, cells from cocultures were liberated with trypsin, counted and the ratios between EC and ASC were determined by flow cytometry analysis (*n* = 3). For all graphs: **P ≤ 0.01, ***P ≤ 0.001.

**Figure 2:**
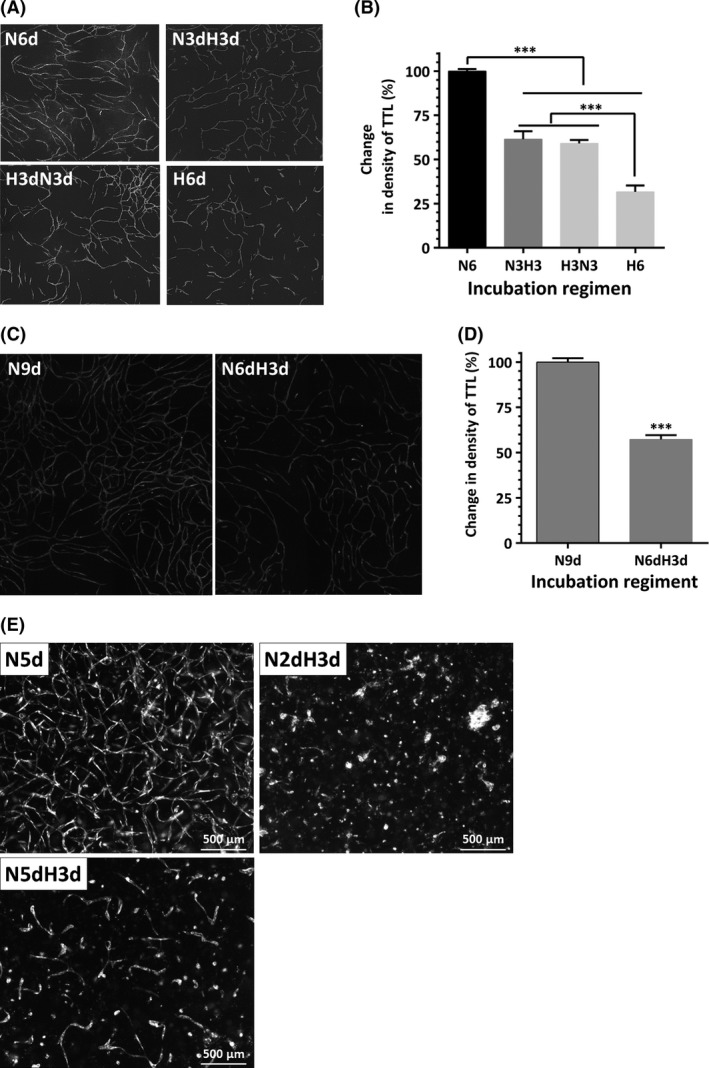
(**A**,** B**) Representative images (**A**) and comparative quantitative analysis (**B**) of VNF (density of total tube length, TTL) of the networks established by CBD‐EC on ASC when cocultures were incubated in normoxia (N6) or at 0.5%O_2_ (H6) for the entire length of experiment (6 days) or when cocultures first were incubated in normoxia for 3 days followed by incubation in hypoxia for 3 days (N3H3), or the other way around (H3dN3d) (*n* = 15–65). (**C**,** D)**, Representative images (**C**) and comparative quantitative analysis (**D**) of VNF of the networks established by CBD‐EC on ASC when cocultures were incubated in normoxia for 9 days (N9) or when cocultures first were incubated in normoxia for 6 days followed by incubation in hypoxia (0.5%O_2_) for 3 days (N6H3) (*n* = 7–8). (**E**), Representative images of vascular networks established by DsRed‐labelled CBD‐EC when cocultured with ASC in collagen/fibrinogen matrix under EBM‐2/5%FBS either in normoxia for 5 days (N5d) or in normoxia for 2 days followed by incubation in hypoxia for 3 days (N2dH3d), or in normoxia for 5 days followed by incubation in hypoxia for 3 days (N5dH3d). For all graphs: ***P ≤ 0.001.

To evaluate the effect of hypoxia on EC vasculogenic activity, EC‐ASC cocultures were incubated in normoxic (21% O_2_) or hypoxic (0.5–5% O_2_) conditions. Analysis performed on day 6 revealed that EC derived from several tissues established dense highly branched vascular networks when incubated in normoxia, as we have previously shown 21 (Fig. 1A). In parallel, cocultures incubated at 5% or 1% O_2_ developed similar (5% O_2_) or slightly less dense (1% O_2_) networks (Fig. 1B). However, remarkably, cocultures exposed to 0.5% O_2_ formed short EC cords, did not assemble into networks and showed a 65% reduction in density of total tube length compared to normoxic cocultures (Fig. 1A and B). This decrease in formation of vascular cords at 0.5% O_2_ was a consistent finding for each tested EC, including those derived from cord blood, umbilical cord vein and retinal and cardiac microvasculature. (Fig. 1A). Analysis of cellular composition of EC‐ASC cocultures at day 6 revealed that while ASC well tolerated hypoxia, less than 25% of CBD‐EC survived (Fig. 1C). Significant and similar drops in densities of vascular networks were also observed when cocultures were exposed for 3 days to 0.5% O_2_ either immediately after plating or after the initial establishment of EC vascular cords at 21% O_2_ (Fig. 2B). However, in both cases, the densities of vascular networks were 50% higher than in cocultures incubated in hypoxia for the entire duration of experiment (Fig. 2A and B). Interestingly, networks allowed to mature during 6 days of EC‐ASC coculture in normoxia showed clear disassembly when exposed to hypoxia for a subsequent 3 days (Fig. 2C and D).

Observations made in this two‐dimensional model of vasculogenesis were strengthened using a complementary three‐dimensional model. In the latter, mixtures of EC and ASC were embedded into fibrin gels and incubated in normoxic conditions for 48 hrs to allow EC and ASC to establish crosstalk in a favourable environment and initiate EC re‐organization into cords; after that cultures were either maintained in normoxia or transferred into hypoxia for a final 3 days of incubation. Cells that were incubated only in normoxia established dense networks, whereas no structures were observed in gels transferred to hypoxia (Fig. 2E). Similar to what was observed in two‐dimensional culture, after development of vascular networks in normoxia for 5 days, transfer to hypoxia for three additional days caused rapid involution of established vascular cords (Fig. 2E).

### Effect of hypoxia on expression of activin A, its inhibitor and receptor

**Figure 3:**
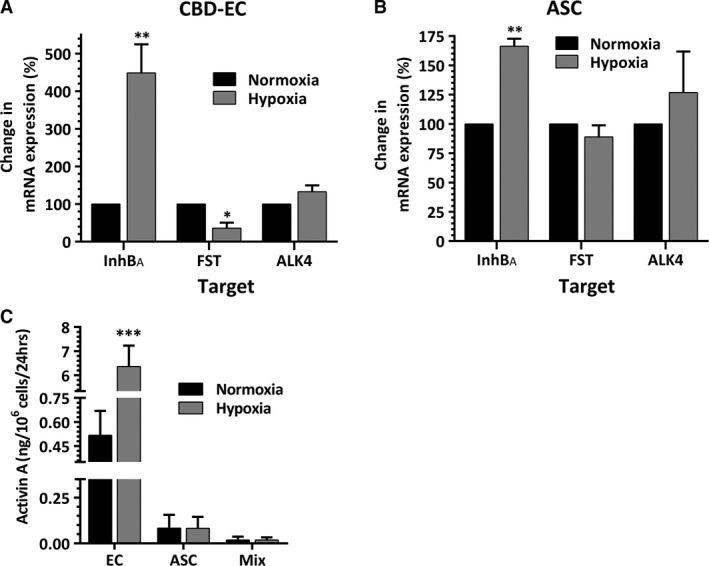
(**A, B**) Effect of hypoxia (0.5%O_2_) on mRNA expression of inhibin B_A_, follistatin and ALK4 by EC (**A**) and ASC (**B**). Expression analysis was performed 24‐hrs post‐hypoxia exposure (*n* = 3–10). (**C**) Effect of hypoxia (0.5%O_2_) on activin A accumulation in the media conditioned by EC, ASC and their cocultures for 48 hrs (*n* = 6–7). For all graphs: *P ≤ 0.05, **P ≤ 0.01, ***P ≤ 0.001.

Exposure of CBD‐EC to 0.5% O_2_ resulted in 4.5‐fold increase in expression of mRNA for inhibin B_A_ (the monomer of activin A), but decrease in mRNA expression of activin A inhibitor follistatin by 65%. The expression level of ALK4 (activin A receptor) mRNA was minimally modulated by hypoxia (Fig. 3A). In parallel, hypoxia led to 1.6‐fold increase in inhibin B_A_ mRNA expression in ASC, but had no effect on expression of follistatin or ALK4 (Fig. 3B). Hypoxia‐induced up‐regulation of inhibin B_A_ mRNA expression in EC translated into 10‐fold increase in activin A accumulation in 48‐hr EC‐CM generated at hypoxia compared to the level produced by normoxic EC (Fig. 3C). In contrast, very low, if any, measurable activin A was observed in media conditioned by ASC at either oxygen level. In concert with that, very low levels of activin A were detected in EC‐ASC‐CM, as ASC represented the majority of cells in the typical coculture model condition.

### Role of Hypoxia Inducible factors in activin A expression

**Figure 4:**
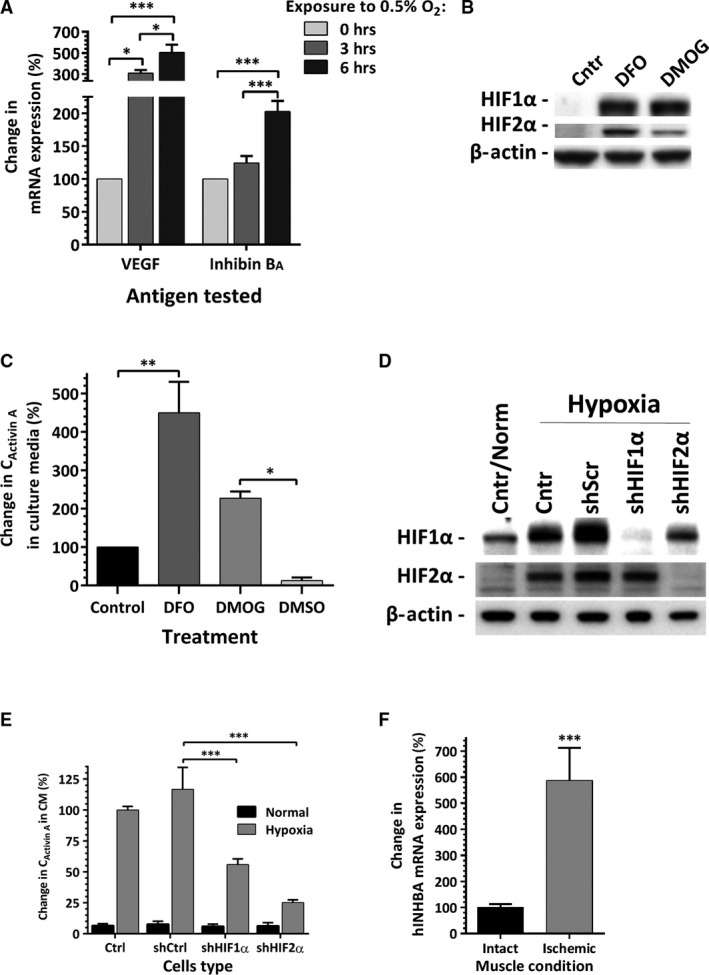
(**A**) Dynamic of VEGF and inhibin B_A_ mRNA expression in EC as a function of time of hypoxia (0.5%O_2_) exposure (*n* = 6–9). (**B**) Analysis of HIF1α and HIF2α protein expressions in EC exposed to control media alone or supplemented with hypoxia mimetics DFO or DMOG (β‐actin served as a control for protein loading). (**C**) Analysis of activin A accumulation in the media condition by EC for 24 hrs while exposed to control media alone (Control) or supplemented with DFO or DMOG (*n* = 3). (**D**) Analysis of HIF1α and HIF2α protein expression in unmodified EC (Cntr) and EC expressing either scrambled, or HIF1α, or HIF2α shRNA after cell exposure to normoxia or hypoxia (0.5% O_2_) for 40 hrs (β‐actin served as a control for protein loading). (**E**) Analysis of activin A accumulation in the media conditioned in normoxia or hypoxia by unmodified EC (Cntr) and EC expressing either scrambled, or HIF1α, or HIF2α shRNA. The level of activin A detected in conditioned media of control EC incubated in hypoxia is expressed as 100%. (**F**) Detection of expression of human inhibin B_A_ mRNA in mouse intact and ischaemic hindlimb muscles twenty‐four hours after receiving local injection of human CBD‐EC. Level of human beta‐actin mRNA was used for normalization. For all graphs: *P ≤ 0.05, **P ≤ 0.01, ***P ≤ 0.001.

Time–course analysis of inhibin B_A_ mRNA expressions in EC was performed to test whether inhibin B_A_ behaves as a direct target of hypoxia inducible factors (HIF). Up‐regulation of VEGF, a known HIF‐regulated factor, was observed after 3 hrs of hypoxia exposure; and a significant increase in inhibin B_A_ expression was detected at the sixth incubation hour (Fig. 4A). To support the hypothesis that inhibin B_A_ expression in EC is HIF regulated, cells were incubated in media containing DFO or DMOG, factors known to mimic hypoxic environment by two complementary mechanisms. EC, exposed to either of these factors for 24 hrs at 21% O_2_, have shown significant intracellular accumulation of HIF‐1α and HIF‐2α (Fig. 4B). In parallel, DFO and DMOG treatment led to 4.5‐fold and 2.3‐fold increases in activin A concentrations in the incubation media respectively, compared to its level in EC‐conditioned control media (Fig. 4C). To strengthen this initial observation, EC stably expressing shRNA for HIF‐1α and HIF‐2α were generated. The efficiency of HIFs silencing was confirmed by exposing EC to hypoxia for 40 hrs followed by analysis of HIFs expression. In control and scrambled shRNA‐transduces, EC hypoxia induced increase in cellular accumulation of both HIF‐1α and HIF‐2α, whereas in EC with silenced HIFs, no detectable levels of corresponding protein were observed (Fig. 4D). As was expected, exposure of control cells to hypoxia led to profound increase in activin A secretion. In parallel, analysis of the media conditioned in hypoxia by HIF‐1α‐ or HIF‐2α‐silenced EC, revealed a decrease in activin A levels by 52% and 78% correspondingly, compared to its level in condition media of scrambled shRNA EC (Fig. 4E).

### Expression of human activin A by EC in ischaemic tissue

To evaluate the effect of *in vivo* ischaemia on inhibin B_A_ expression by human CBD‐EC, cells were locally injected into the ischaemic and corresponding contralateral intact (non‐ischaemic) tibialis anterior muscle 3 hrs after surgically created ischaemia in mouse hindlimbs. Analysis of human‐specific inhibin B_A_ expression in the muscles harvested twenty‐four hours post‐injection revealed a nearly sixfold increase in its level in human EC due to ischaemia exposure within the skeletal muscle (Fig. 4F).

### Role of activin A in EC survival and VNF

**Figure 5:**
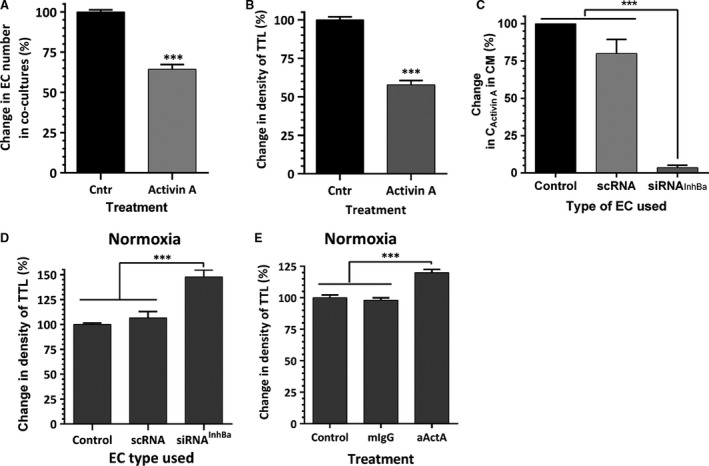
(**A**) Analysis of CBD‐EC survival in the networks established by CBD‐EC on ASC monolayer during incubation in control media alone (Cntr) or supplemented with 25 ng/ml of activin A for 3 days (*n* = 18). (**B**) Comparative analysis of the degree of vascular network formation (density of total tube length, TTL) by EC‐ASC cocultures when cells were incubated in EBM‐2/5%FBS media alone (Cntr) or in the presence of 25 ng/ml of activin A for the first 3 days of 6‐day incubation (*n* = 12–18). (**C**), Analysis of activin A accumulation in the media conditioned by either unmodified CBD‐EC or CBD‐EC transfected with either inhibin B_A_ silencing RNA (siRNA) or with scrambled RNA (scRNA) for twenty‐four hours. Media were collected 48‐hrs post‐transfection (*n* = 6–10). (**D**) Comparative analysis of vascular networks formed by either unmodified CBD‐EC or CBD‐EC transfected with either inhibin B_A_ silencing RNA (siRNA) or with scrambled RNA (scRNA) when in coculture with ASC (*n* = 7–10). (**E**) Comparative analysis of vascular network formation by EC‐ASC cocultures incubated in control media alone or in the presence of 10 μg/ml of neutralizing activin A or isotype control IgG. For all graphs: ***P ≤ 0.001.

As we previously reported 24 and as shown in Figures 5A and B, challenging normoxic EC‐ASC cocultures with activin A for the first 3 days of incubation caused a 35% loss of EC and diminished the ultimate density of vascular networks by 40%. Conversely, blocking expression of activin A in EC by transfecting cells with inhibin B_A_ siRNA (97% efficiency of knockdown assessed by ELISA, Fig. 5C) led to a 47% increase in network density in normoxia (Fig. 5D). Similarly, activin A neutralizing IgG, added to incubation media, induced a 25% increase in vessel density compared to control or isotype control IgG‐containing cultures (Fig. 5E).

**Figure 6:**
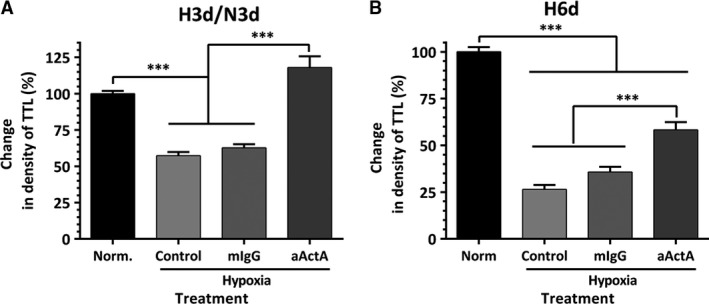
(**A**) Comparative analysis of vascular network formation (density of total tube length, TTL) by EC‐ASC cocultures incubated in normoxia in control media or in hypoxia in control media alone or supplemented with either 10 μg/ml of neutralizing activin A or isotype control IgG for 3 days, followed by media exchange to control media and an additional 3‐day incubation in normoxia (*n* = 21–31). (**B**) Comparative analysis of vascular network formation by EC‐ASC cocultures incubated in normoxia in control media or in hypoxia in control media alone or supplemented with either 10 μg/ml of neutralizing activin A or isotype control IgG for 6 days (*n* = 14–15). For all graphs: ***P ≤ 0.001.

More prominent protective effects of activin A IgG were observed when EC‐ASC cocultures were exposed to hypoxia. While exposure of EC‐ASC cocultures to hypoxia for the first 3 days of a 6‐day incubation caused a 35% drop in vessel density, the addition of activin A IgG to the incubation media maintained the VNF at the level of cocultures kept in normoxia throughout the experiment (Fig. 6A). Furthermore, while exposure of cocultures to hypoxia for 6 days caused a 75% decrease in vessel density, the addition of activin A IgG even only for the first 3 days of incubation diminished the disruptive effect of hypoxia by more than 50% (Fig. 6B).

**Figure 7:**
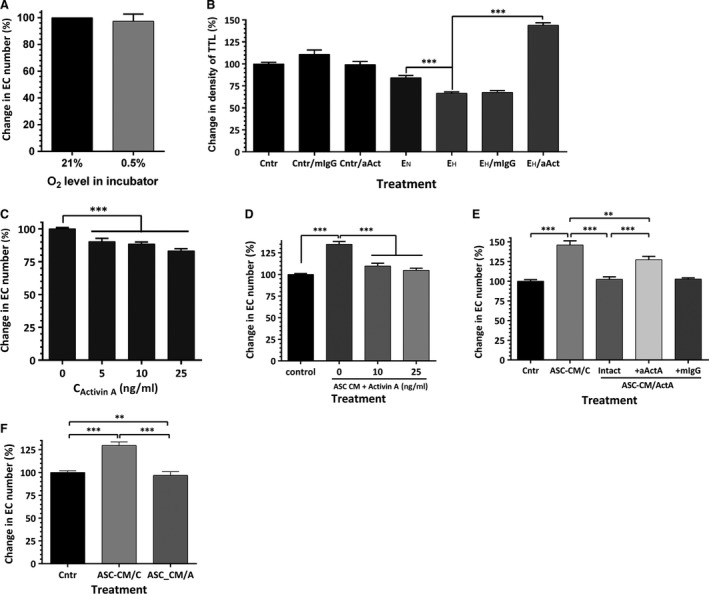
(**A**) Analysis of hypoxia effect on survival/proliferation of CBD‐EC after 3‐day exposure (*n* = 7). (**B**) Comparative analysis of vascular network formation (density of total tube length, TTL) by EC‐ASC cocultures after 6‐day incubation in either control media (Cntr), or in media conditioned by CBD‐EC which were cultured in standard conditions (E_N_) or in hypoxia (E_H_). Additionally, cocultures were exposed to control media or E_H_‐conditioned media, which, prior to be added to cocultured, were pre‐treated with either activin A neutralizing or isotype control IgG (*n* = 3–6). (**C**) Analysis of CBD‐EC survival/proliferation in response to 3‐day treatment with 5–25 ng/ml activin A (*n* = 11–44). (**D**) Analysis of mitogenic response of CBD‐EC to media conditioned by ASC in hypoxia and the effect of activin A (10–25 ng/ml) on its modulation (*n* = 16–32). (**E**) Analysis of CBD‐EC mitogenic response to 48‐hr conditioned media produced by ASC in control media alone or in the presence of 25 ng/ml activin A (ASC‐CM/ActA). CBD‐EC were exposed to ASC‐CM/ActA unmodified or pre‐treated with activin A neutralizing or isotype control IgG (*n* = 8–16). (**F**) Analysis of mitogenic response of CBD‐EC to 24‐hr conditioned media produced by unstimulated ASC and by ASC after 48‐hr pre‐treatment with 25 ng/ml of activin A (*n* = 12–23). For all mitogenesis tests, cell counts were performed after 3 days of treatment exposure. For all graphs: **P ≤ 0.01, ***P ≤ 0.001.

To test whether the decrease in VNF in hypoxia was primarily due to downstream effects of a change in the EC secretome, EC confluent monolayers were incubated in standard conditions or at 0.5% O_2_ for 48–72 hrs for media conditioning. Remarkably, analysis of cell number at the end of incubation revealed no differences in cell numbers between two incubation conditions, suggesting that EC, when cultured alone, tolerated hypoxia well (Fig. 7A). Seventy‐two hour EC CMs were collected, concentrated 10‐fold, then diluted in ratio 2:3 with EBM‐2/5%FBS and applied on EC‐ASC cocultures for the first 3 days of incubation. Exposure of cocultures to media conditioned by EC in hypoxia (E_H_) caused a 24% drop in vascular density, whereas media from control EC (E_N_) had no effect (Fig. 7B). However, when E_H_ CM were pre‐treated with activin A IgG, a substantial increase in density of vascular networks (above the level of control cultures) was observed.

To evaluate the mechanism through which activin A manifests this activity, EC, while in hypoxia, were exposed to 5–25 ng/ml of activin A for 3 days. Analysis revealed that activin A caused up to 16% decrease in EC numbers as a result of treatment (Fig. 7C). Previously, we have shown that media conditioned by ASC promote EC survival/proliferation in standard culture conditions 23, 24, 25. This prior observation is further supported here with the finding of a similar protective effect of ASC‐CM generated in hypoxic environment on EC incubated in hypoxia: ASC‐CM increased EC numbers by 35% in comparison with control cultures (Fig. 7D). However, addition of activin A (10–25 ng/ml) abolished most of the positive effect of ASC‐CM, suggesting that activin A plays a dominant negative role in modulating EC bioactivity by ASC secretome.

To test whether activin A also modifies ASC secretome, ASC‐conditioned media were generated in the presence of 25 ng/ml activin A for 48 hrs. As expected, control ASC‐CM increased the EC number by 46%, whereas media from activin A‐treated ASC had no effect (Fig. 7E). To eliminate the effect of residual recombinant activin A, conditioned media were pre‐treated with activin A neutralizing IgG prior to applying it on EC (Fig. 7E). Such pre‐treatment led to increase in cell number by only 27% (compared to the effect of media pre‐treated with isotype control IgG), suggesting that either exogenous or ASC‐derived activin A was responsible only for approximately one‐half of the observed effect, whereas the remainder of the effect is attributed to additional downstream changes in ASC‐CM composition as a result of ASC exposure to activin A (Fig. 7E). This was further supported by the finding that media conditioned by ASC after activin A stimulus withdrawal were also unable to promote EC survival/proliferation (Fig. 7F).

## Discussion

Local injection of vasculogenic cell mixtures of endothelial and mesenchymal progenitor cells has been proposed as an attractive therapeutic approach to promote restoration of blood flow in ischaemic tissues 26, 27, 28, 29. However, at the site of injection, these cells are immediately exposed to unfavourable elements of the ischaemic environment, including low oxygen level, inflammatory cytokines (TNF‐α, IL‐1β, IL‐6) and reactive oxidative species, which collectively cause substantial loss of cells, especially EC. Jointly, these factors diminish the therapeutic effectiveness of donor cells. Understanding cellular responses to these challenges will allow to improve prediction of cell fate after delivery and to suggest potential targets for interference to improve cell survival and tissue revascularization. While the findings of this study are particularly relevant to the bioactivity of the cells used for vascular cell therapies, some outcome may be extrapolated to the responses of the native tissue vasculature.

Most prior studies of the effects of hypoxia have assessed either responses of the cell monocultures to hypoxia, an approach that does not account for real‐time crosstalk between the cells in the niche, or used coculture models in which cells of different origins were spatially separated and thus did not account for heterotypic juxtacrine and proximate paracrine signalling. The majority of these studies have shown that hypoxia induces vasculo‐ and angiogenic programmes. Pertinent to the current study, hypoxia boosts mitogenic activity of ASC 30, 31 and up‐regulates expression of multiple cytokines, including VEGF, basic fibroblast growth factor, interleukin‐6, angiopoietin‐1, brain‐derived neurotrophic factor and stromal cell‐derived factor‐1 23, 30, 31, 32. Media conditioned by ASC in hypoxia promote EC survival and proliferation to a greater degree than media conditioned in normoxia 23, 30 and stimulate EC cord formation on Matrigel 33. However, EC responses to hypoxia are controversial. While some prior studies suggest that hypoxia decreases EC survival, proliferation and tubulogenesis 34, others have demonstrated an increase in EC tubulogenesis 35, 36.

To explore these critical effects of hypoxia in a context closer to that of a physiological niche, we employed here a coculture model of EC with ASC 19. ASC, which are progenitors with many phenotypical and functional characteristics of mural cells (pericytes and smooth muscle cells) 19, 25, 26, represent an attractive cell source providing an EC niche in which to study the process of therapeutic vasculogenesis. The EC‐ASC coculture model was previously established by us to facilitate dissection of the mechanisms of heterotypic cell–cell interactions leading to vessel formation. While we have documented that ASC strongly support EC vasculogenesis in normoxia, the response of these cocultures to low oxygen levels was not previously explored.

Previous studies have explored a wide range of oxygen concentrations (0.5–5%) considered to represent hypoxia. The fact that various tissues are exposed to widely differing physiological levels of oxygen tension 37 makes it challenging to define appropriate cell‐type‐specific hypoxic conditions. Therefore, we assessed EC survival and organization into vascular networks at several O_2_ levels. EC cultivated on ASC were tolerant to decreases in oxygen levels down to 1%, with neither decrease nor increase in degree of vasculogenesis, while a detrimental effect was observed at 0.5% O_2_ (Fig. 1). Similar effects were observed in three‐dimensional vasculogenesis model, where EC and ASC are initially not in direct contact and exclusively rely on paracrine rather than direct contact communication (Fig. 2E).

D. Hutton and W. Grayson reported a study, in which they demonstrated that VNF was already affected at 2% O_2_ 38. This discrepancy in EC responses to hypoxia between the studies could be explained by different equipment used to establish and control low oxygen conditions. However, a remarkable difference is that in the present study, the exposure of fully established vascular networks (following 5 or 6 days of incubation at 21% O_2_) to 0.5% O_2_ caused network degradation (Fig. 2C), whereas in the study by Hutton and Grayson, placing established networks into 0.2% O_2_ did not cause degradation 38. Also, the authors attributed lower vessel density in hypoxic cultures to decrease in EC proliferation, whereas in our study, the decrease in vessel density was attributed to net EC loss (Fig. 1C). These discrepancies in findings will need further evaluation.

The key finding of this study is that EC respond to hypoxia by up‐regulating activin A secretion (Fig. 3C), which was further strengthened by the results of similar EC response to ischaemic environment *in vivo* (Fig. 4F). These findings extend the observations by Manalo *et al*., that human pulmonary artery EC up‐regulate inhibin B_A_ mRNA when exposed to 1% O_2_ or transfected with a constitutively active HIF‐1α 39. The facts that 6 hrs of hypoxia exposure was sufficient to up‐regulate inhibin B_A_ mRNA in EC (Fig. 4A), and its expression is modulated by hypoxia mimetics DFO and DMOG (Fig. 4C) suggest that inhibin B_A_ expression is under control of HIFs. Experiments with targeted silencing of HIF1α and HIF2α clearly showed that both HIFs are involved in regulation of activin A secretion (Fig. 4E).

Up‐regulation of activin A production has been reported for multiple ischaemic conditions, including acute kidney injury, myocardial infarction and brain insult 1, 2, 3, 4, 5, 6, 7, 8. In many cases, up‐regulation of activin A concurs with decrease in expression of its inhibitor follistatin 7, 8. Activin A activities are organ‐specific; while in ischaemic brain, it has tissue‐protective effects 7, in ischaemic kidney, it produces detrimental effect 8, 40 and in myocardium, its effects are controversial. While some have reported that activin A promotes recovery of ischaemic myocardium 5, others have claimed that blocking activin A diminishes ischaemia/reperfusion‐induced myocardial infarction 41. Serum levels of activin A are elevated in patients with heart failure, positively correlating with disease severity 4. Interestingly, the induction of activin A has been reported only in functional cells, such as kidney tubular cells 8, cardiomyocytes 4 or neurons 7, 20, whereas no data are available of its expression in the vasculature of affected tissues.

We previously reported several critical roles of activin A in EC‐ASC communication, including a primary role in ASC differentiation from progenitor towards mural (smooth muscle cell) phenotype 19 as well as in shifting ASC secretome from pro‐angiogenic towards angiostatic 24. These complementary activin A activities both promote formation and maintenance of mature stable vascular structures 21. It is important to mention that up‐regulation of activin A in these prior studies was observed exclusively in ASC, which was dependent on EC‐ASC juxtacrine communication.

The analyses of EC‐ASC communication in hypoxia during this study suggested another prospective role of activin A in crosstalk between these partner cells. Previously, we have shown that blocking activin A secretion by ASC led to denser vascular networks 24. This observation was extended here by demonstration of a similar effect when secretion of activin A was blocked in EC (Fig. 5C). As we reported previously, in the model used here, the secretion of activin A by ASC was induced between days 3 and 4 19. Therefore to specifically assess the effects of EC‐derived activin A on EC‐ASC vasculogenesis, cocultures were treated with activin A IgG only for the first 3 days of incubation while exposed to 0.5% O_2_. While hypoxia induces perturbations in secretion of multiple factors in both cell types, the fact that blocking activin A was sufficient to eliminate or lower the undesirable effect of hypoxia suggests a key role of activin A in observed effects (Fig. 6A and B). This is further strengthened by the finding that exposure of EC‐ASC cocultures to EC‐CM produced in hypoxia decreases vascular density by 25% when compared with effect of EC‐CM produced in normoxia (Fig. 7B). Interestingly, pre‐treatment of EC‐CM with activin A IgG unleashed its strong pro‐vasculogenic activity, proving that if it was not for the presence of activin A, EC secretome would possess a strong vasculogenic phenotype (Fig. 7B).

Vasculogenesis in EC‐ASC coculture model depends on multiple factors, secreted by ASC (*e.g*. VEGF, hepatocyte growth factor, SDF‐1), which are responsible for EC survival, proliferation, migration and tubulogenesis 42, 43, 44. The central question that arises is whether activin A affects EC bioactivity directly or indirectly by modifying activity of ASC. The fact that recombinant activin A completely blocks the mitogenic effect of ASC‐CM on EC proliferation under hypoxic conditions (Fig. 7C and D) suggests that activin A has a direct effect on EC. In parallel, we have previously reported that activin A down‐regulates secretion of hepatocyte growth factor, SDF‐1 and angiopoietin‐1 in ASC as well as bioavailability of VEGF in cocultures 24, therefore most likely reducing the potency of ASC secretome to support EC survival and proliferation. This hypothesis is supported by finding that ASC‐CM generated under hypoxic conditions during or post‐exposure to activin A partially or completely lost the ability to support EC survival/proliferation (Fig. 7E and F).

In conclusion, the robust production of activin A, by EC in response to significant decrease in oxygen tension, results in loss of EC *via* direct and indirect mechanisms and consequently profound inhibition of the development of new vessels; this effect can be blocked by silencing activin A activity. We speculate that selective interference with activin A signalling will provide a novel practical strategy to preserve vasculature, compromised by ischaemia and will improve ischaemic tissue revascularization by locally injected vasculogenic cell mixtures, composed of EC and MSC (including ASC).

## Conflict of interest

The authors confirm that there is no conflict of interests.
